# Respiratory Muscle Training Improves Diaphragm Citrate Synthase Activity and Hemodynamic Function in Rats with Heart Failure

**DOI:** 10.21470/1678-9741-2017-0002

**Published:** 2017

**Authors:** Rodrigo Boemo Jaenisch, Mariane Bertagnolli, Audrey Borghi-Silva, Ross Arena, Pedro Dal Lago

**Affiliations:** 1 Laboratory of Experimental Physiology, Post-Graduation Program in Health Sciences, Universidade Federal de Ciências da Saúde de Porto Alegre (UFCSPA), Porto Alegre, RS, Brazil.; 2 Cardiopulmonary Physiotherapy Laboratory, Physiotherapy Department, Universidade Federal de São Carlos (UFSCAR), São Carlos, SP, Brazil.; 3 Cardiovascular Clinical Research Facility, Division of Cardiovascular Medicine, University of Oxford, Oxford, United Kingdom.; 4 Department of Physical Therapy, College of Applied Health Sciences, University of Illinois at Chicago, Chicago, IL, USA.

**Keywords:** Myocardial Infarction, Exercise, Citrate Synthase, Heart Failure, Breathing Exercises, Hemodynamics

## Abstract

**Introduction:**

Enhanced respiratory muscle strength in patients with heart failure positively alters the clinical trajectory of heart failure. In an experimental model, respiratory muscle training in rats with heart failure has been shown to improve cardiopulmonary function through mechanisms yet to be entirely elucidated.

**Objective:**

The present report aimed to evaluate the respiratory muscle training effects in diaphragm citrate synthase activity and hemodynamic function in rats with heart failure.

**Methods:**

Wistar rats were divided into four experimental groups: sedentary sham (Sed-Sham, n=8), trained sham (RMT-Sham, n=8), sedentary heart failure (Sed-HF, n=7) and trained heart failure (RMT-HF, n=7). The animals were submitted to a RMT protocol performed 30 minutes a day, 5 days/week, for 6 weeks.

**Results:**

In rats with heart failure, respiratory muscle training decreased pulmonary congestion and right ventricular hypertrophy. Deleterious alterations in left ventricular pressures, as well as left ventricular contractility and relaxation, were assuaged by respiratory muscle training in heart failure rats. Citrate synthase activity, which was significantly reduced in heart failure rats, was preserved by respiratory muscle training. Additionally, a negative correlation was found between citrate synthase and left ventricular end diastolic pressure and positive correlation was found between citrate synthase and left ventricular systolic pressure.

**Conclusion:**

Respiratory muscle training produces beneficial adaptations in the diaphragmatic musculature, which is linked to improvements in left ventricular hemodynamics and blood pressure in heart failure rats. The RMT-induced improvements in cardiac architecture and the oxidative capacity of the diaphragm may improve the clinical trajectory of patients with heart failure.

**Table:** 

Abbreviations, acronyms & symbols				
CABG	= Coronary artery bypass graft		MI	= Myocardial infarction
CS	= Citrate synthase		VO_2_	= Peak oxygen consumption
HF	= Heart failure		PPCs	= Postoperative pulmonary complications
HR	= Heart rate		RMT	= Respiratory muscle training
LV	= Left ventricle		RV	= Right ventricle
LVEDP	= Left ventricular end-diastolic pressure		SAP	= Systolic arterial pressure
LVSP	= Left ventricular systolic pressure		SD	= Standard deviation
MAP	= Mean arterial pressure		STICH	= Surgical Treatment for Ischemic Heart Failure

## INTRODUCTION

Heart failure (HF) is related to reduced cardiac output as well as blood flow to the skeletal muscle, which impairs cell aerobic metabolism, particularly during physical exertion^[1]^. The inability of the heart to supply adequate amounts of blood to meet the metabolic needs of peripheral tissues^[2]^, including the diaphragm muscle, is likely a key pathophysiological mechanism of HF.

Previous studies using a HF model induced by myocardial infarction (MI) demonstrated reduced contractility, tension and structural abnormalities in the diaphragmatic musculature^[3]^. From a clinical perspective, patients with HF often present with inspiratory muscle weakness^[4]^ and peripheral skeletal muscle dysfunction^[5]^, which are important contributors to exercise intolerance and culminate in poor prognosis^[4,5]^. In addition, peripheral muscle abnormalities in HF, such as impaired skeletal muscle energy metabolism, mitochondrial dysfunction, fiber-type transition and atrophy, play an important role in exercise intolerance^[1]^, which seems to be associated with the genesis of the pathophysiological mechanism. Alterations in muscle energy metabolism related to mitochondrial dysfunction has been assessed through measurement of citrate synthase (CS) activity, one of the first enzymes participating in the citric acid cycle and cellular oxidative metabolism, also reflecting mitochondrial content in muscle tissue^[2,6]^.

In the experimental model of HF, we previously demonstrated that respiratory muscle training (RMT) promoted improvements in cardiopulmonary function^[7]^; however, the potential effect of RMT on metabolic adaptations in the diaphragm is not fully understood. To this end, we conducted a RMT protocol in an experimental model of HF induced by MI in rats; hemodynamic function and CS activity were assessed. Our hypothesis was that the 6-week RMT protocol in rats could increase diaphragm oxidative metabolism, assessed by the CS activity measurement, leading to hemodynamic function improvement.

Coronary artery bypass graft (CABG) surgery has demonstrated good potential in promoting benefits related to cardiovascular mortality^[8]^. Evidence has demonstrated that preoperative RMT reduced the incidence of postoperative pulmonary complications (PPCs) and duration of postoperative hospitalization in patients at high risk of developing a pulmonary complication undergoing CABG surgery. We believe that the present study could contribute to understand these potential mechanisms^[9]^.

## METHODS

### Animals

All of the procedures outlined in this study were approved by the Ethics Research Committee of the Universidade Federal de Ciências da Saúde de Porto Alegre (UFCSPA) (protocol 712/08). Thirty-six male Wistar rats (200 to 250 g; 90 days of age) obtained from the Animal Breeding Unit of the UFCSPA were housed under standard conditions (food and water *ad libitum*, 12:12-h light-dark cycle; 22ºC).

### Experimental Procedures

#### Myocardial Infarction Surgery

The animals were anesthetized with xylazine (12 mg/kg i.p.) and ketamine (90 mg/kg i.p.), intubated and artificially ventilated. For MI, ligation of the left coronary artery was performed, as well as sham operations as previously described^[7,10,11]^. After the surgery, the rats received a single injection of monofenew (0.05 ml/100 g) and gentamicin (0.05 ml/100 g).

Subsequently to MI or sham surgery, rats were followed for 4 weeks (time for HF development)^[7,10]^ and were allocated into four experimental groups: sedentary sham rats (Sed-Sham; n=8), trained sham rats (RMT-Sham; n=8), sedentary HF rats (Sed-HF; n=7), or trained HF rats (RMT-HF; n=7).

### RMT Protocol

Rats assigned to the RMT groups were submitted to a 5-day adaptation protocol, as previously described. The RMT protocol began in the 5^th^ week post-MI or sham surgery. It comprised 30 min/day, 5 days/week, for 6 weeks. The progress of the protocol was achieved by a progressive increase in load resistance, by reducing the internal diameter of the hole through which the animal breathed. During the first week of the training, the orifice at the inspiratory port was set at an internal diameter of 0.8 mm and was progressively decreased, reaching after 2 weeks of training a final internal diameter of 0.3 mm (maximal resistance), as previously described^[7,12]^. Bisschop et al.^[12]^ demonstrated that, in this protocol, the inspiratory load imposed to animals trained was equivalent to a resistance of 0.7 cmH_2_O/ml/s at flow rate of 5 ml/s (with an internal diameter of 0.8 mm) and a resistance of 18.4 cmH_2_O/ml/s at a flow rate of 5 ml/s (with an internal diameter of 0.3 mm).

### Cardiac Hemodynamic Evaluation

After the RMT (RMT groups) or control (sedentary groups) period, the rats were placed general anesthesia as previously described and the arterial catheter (PE-50, 0.5 mm ID, Biocorp) was inserted into the right carotid artery. A strain-gauge pressure transducer (P23 Db, Gould Statham, USA) was used for direct hemodynamic measurements. Signals were passed through a preamplifier (Hewlett-Packard 8805, Puerto Rico) and were delivered to a microcomputer equipped with an analog-to-digital converter board (CODAS, 1 kHz, Dataq Instruments, USA). The arterial pressure was recorded first during a 5-min period. Then, the catheter was positioned inside the left ventricle (LV), and the pulse wave was monitored using the typical graphic registration of ventricular pressure and recorded for 5 min. These data were used to determine mean arterial pressure (MAP), heart rate (HR), left ventricular systolic pressure (LVSP), left ventricular maximum change in pressure over time (+dP/dt_max_), left ventricular minimum change in pressure over time (-dP/dt_max_), and left ventricular end-diastolic pressure (LVEDP)^[7,11]^.

### MI and HF Characterization

All animals were sacrificed by anesthetic overdose (thiopental 80 mg/kg i.p.), and the heart, lungs, liver, and diaphragm were removed and weighed. The right ventricle (RV) and LV were dissected and weighed. LV was placed in 10% formaldehyde for a minimum of 3 days before being cut into two equal transverse sections. These sections were embedded in paraf?n for subsequent analysis of the infarct size. The percentage of the infarcted area was determined as described previously^[7,10]^. The heart weight-to-body weight ratio (HW/BW), LV/BW, and RV/BW values were determined. Lungs and liver were dehydrated (80°C) for 48 h and then weighed again to evaluate the water percentage^[7]^.

### Diaphragm Collection and Measurement of CS Activity

A laparotomy and thoracotomy were performed to allow the complete excision of the diaphragm. The right and left costal diaphragm muscle were weighed and immediately snap-frozen in liquid nitrogen, and stored at -80°C for subsequent analysis.

CS activity, an index of oxidative capacity, was determined in diaphragm muscle by measuring with 5,5-dithiobis-(2-nitrobenzoic acid; DTNB), as described by Alp et al.^[13]^. The absorption was read spectrophotometrically at 412 nm, and the results are expressed as nmol/min/mg protein.

### Statistical Analysis

The mean values and the standard deviation (± SD) were calculated for all the analyzed data. The Kolmogorov-Smirnov normality test was performed. A two-way ANOVA compared the effects between groups (HF or Sham) and intervention (RMT or Sed), followed by the Tukey *post hoc* test. Pearson's correlation analysis was performed to test associations. A *P*<0.05 was considered statistically signi?cant. The GraphPad Prism 6 program (GraphPad Software, CA, USA) was used for the data analysis.

## RESULTS

### Mortality, Morphological Characteristics, Pulmonary and Hepatic Congestion

Overall mortality during and after MI was 33%, with no deaths in the sham groups. The initial body weights and final body weights were similar among the four groups in both the pre- and post-training period. Additionally, there were no differences in the infarcted area between the HF groups.

The Sed-HF group presented pulmonary congestion when compared with sham groups; however, RMT groups have less pulmonary congestion (*P*<0.0001 for training effect). There was no difference in hepatic congestion among groups. The HW/BW and RV/BW were higher in the Sed-HF group compared with sham groups. RMT-HF group showed decrease RV hypertrophy (*P*<0.0001 for training effect); although no difference was observed in the LV hypertrophy with RMT (all of these data are summarized in Table 1).

**Table 1:** Morphological characteristics, infarct area and lung and hepatic congestion of sham-operated groups and rats with left ventricular dysfunction.

Groups	Initial body weight, g	Final body weight, g	Infarcted area, %	HW/BW, mg/g	LV/BW, mg/g	RV/BW, mg/g	Pulmonary congestion, %	Hepatic congestion, %
Sed-Sham	217±12	323±32	__	2.67±0.25†	2.17±0.3	0.43±0.16	69.73±1.15	72.17±0.74
RMT-Sham	211±8	302±30	__	2.46±0.97*	1.97±0.77	0.49±0.14	67.98±4.75	72.85±0.99
Sed-HF	220±7	314±20	43.10+-4	3.46±0.4**	2.58±0.2	1.05±0.22§	76.73±1.39§	72.83±1.23
RMT-HF	218±13	314±30	45.12±9	3.34±0.54	2.52±0.43	0.72±0.22	68.23±4.68	71.64±1.93

Values are means ± SD. Groups were compared by the two-way ANOVA and Tukey post hoc tests. Sedentary sham rats (Sed-Sham; n=8); respiratory muscle training sham rats (RMT-Sham; n=8); sedentary heart failure rats (Sed-HF; n=7); respiratory muscle training heart failure rats (RMT-HF; n=7). HW/BW=heart weight-to-body weight ratio; LV/BW=left ventricle-to-body weight ratio; RV/ BW=right ventricle-to-body weight ratio.

* *P*<0.05 compared with Sed-HF and RMT-HF

† *P*<0.05 compared with RMT-HF.

** *P*<0.01 compared with Sed-Sham.

§ *P*<0.01 compared with Sed-Sham, RMT-Sham and RMT-HF.

### Hemodynamic Alterations with RMT

**Fig. 1:**
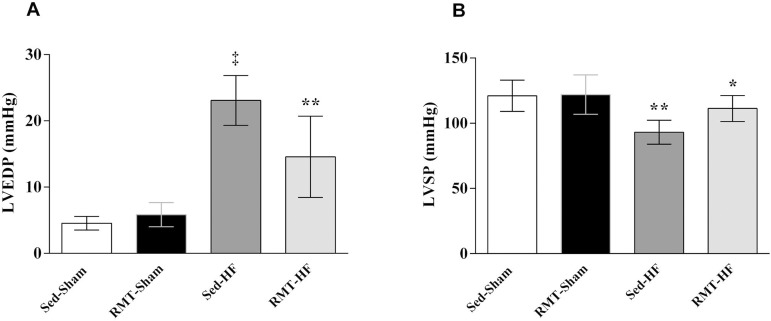
Values are means ± SD. Two-way ANOVA and Tukey post hoc test. Sedentary sham rats (Sed-Sham; n=8); respiratory muscle training sham rats (RMT-Sham; n=8); sedentary heart failure rats (SedHF; n=7); respiratory muscle training heart failure rats (RMT-HF; n=7). A=LVEDP, LV end-diastolic pressure. ‡P<0.0001 compared with Sed-Sham, and RMT-Sham; **P<0.001 compared with Sed-HF, Sed-Sham, and RMT-Sham. B=LVSP, LV systolic pressure. **P<0.001 compared with Sed-Sham, and RMT-Sham.

The Sed-HF group had higher LVEDP; however, the LVSP, +dP/dt_max_ and −dP/dt_max_ were lower, characterizing the HF state in this post-MI model. RMT in HF rats reduced the LVEDP (*P*<0.001 for group, *P*<0.05 for training, and *P*<0.001 for interaction effects, Figure 1A) and increased LVSP (*P*<0.0001 for group, and *P*<0.05 for training effects, Figure 1B). +dP/dt_max_ was similar between the RMT-HF group and sham groups.

Whereas MAP and systolic arterial pressure (SAP) were lower in the Sed-HF group, characteristics of HF, RMT prevented blood pressure drops in the HF group (*P*<0.05 for training, and *P*<0.05 for interaction effects; *P*<0.01 for group, and *P*<0.01 for interaction effects, respectively). There were no differences in heart rate between the groups.

### CS Activity

**Fig. 2:**
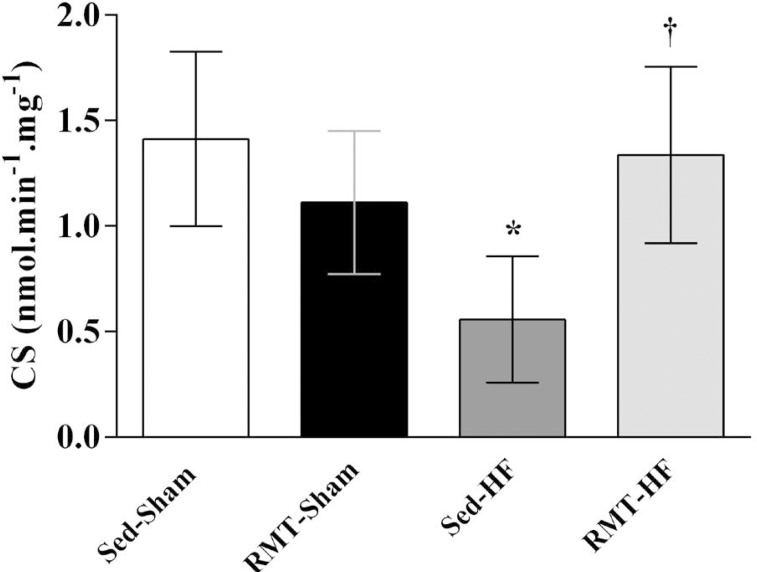
Values are means ± SD. Two-way ANOVA and Tukey post hoc test. Sedentary sham rats (Sed-Sham; n=6); respiratory muscle training sham rats (RMT-Sham; n=6); sedentary heart failure rats (Sed-HF; n=4); respiratory muscle training heart failure rats (RMT-HF; n=4). CS=citrate synthase. *P<0.05 compared with Sed-Sham; †P<0.05 compared with Sed-HF.

CS activity, a marker of mitochondrial content tissue, was lower in Sed-HF rats (Figure 2). RMT increased CS activity in the diaphragm in rats with HF (Figure 2).

**Fig. 3:**
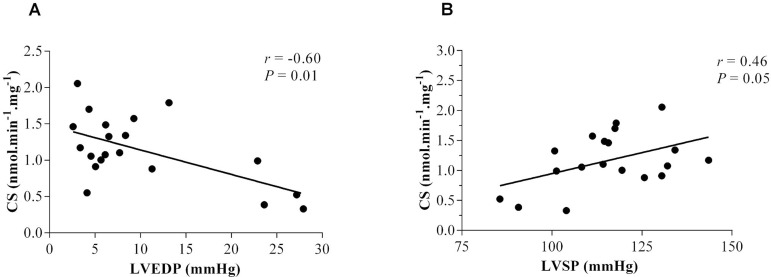
Correlations between citrate synthase and hemodynamic function parameters in sham rats and HF rats. A=CS, citrate synthase; LVEDP, LV end-diastolic pressure. r=–0.60, P<0.01 B=CS; LVSP, LV systolic pressure. r=0.46, P<0.05.

To test the effects of RMT on the association between oxidative capacity and hemodynamic function, we tested the correlation between CS activity and hemodynamic variables. Interestingly, CS alterations in the diaphragm due to HF and RMT demonstrated a significant correlation with hemodynamic function in rats. A negative correlation was found between CS and LVEDP (r=-0.60, *P*=0.01) (Figure 3A) and a positive correlation was found between CS and LVSP (r=0.46, *P*=0.05) (Figure 3B).

## DISCUSSION

In the present study, it was demonstrated that: 1) RMT in rats with HF improved LV hemodynamics, as demonstrated by LV pressures, contractility and relaxation, as well as decreased RV hypertrophy and lung congestion; 2) CS activity was decreased in the diaphragm of the HF rats, and enhanced by RMT; and 3) Correlations were found between CS activity and hemodynamic function parameters, as demonstrated by a negative correlation between CS and LVEDP and a positive correlation between CS and LVSP.

Our results highlight the particular link between hemodynamic dysfunction and the oxidative impairment of diaphragm muscle. An increase in LVEDP and decrease in LVSP were associated with CS activity reduction. Such a link may directly contribute to the establishment and progression of lung congestion and RV overload in response to HF. On the other hand, RMT clearly promotes the overall improvement of cardiac hemodynamics, peripheral muscle oxidative metabolism and mitochondrial function, here reflected by CS activity. This supports the notion that RMT is a viable strategy to positively alter RV and lung alterations as consequence of HF progression.

From a clinical perspective, RMT was shown to improve many outcomes related to the progression and complexity of HF^[14]^, which include dyspnea, peripheral muscle sympathetic nervous activity, oxygen uptake efficiency, circulatory power, recovery oxygen kinetics, indices of cardiac performance, peak oxygen consumption (VO_2_) and quality of life^[14]^. Furthermore, RMT decrease PPC of postoperative atelectasis, pneumonia, duration of hospital stay^[15]^, and improves functional capacity submaximal and inspiratory muscle strength^[16]^ in patients undergoing cardiac surgery. However, the effect of RMT on metabolic adaptations in the diaphragm is not fully understood.

Experimentally, our group has previously demonstrated RMT in HF rats improved cardiovascular parameters, sympathetic and parasympathetic modulation, baroreflex gain and respiratory mechanics^[7]^. However, we did not assess the effects of RMT on oxidative capacity in the diaphragm in rats with HF, which could explain some potential physiologic muscular mechanisms.

Few studies that directly assessed intrinsic skeletal muscle oxidative capacity in HF models reported a decrease in both oxidative and glycolytic muscles^[17]^. Specifically, CS activity was decreased in the experimental model^[17]^ and clinical studies with HF^[18]^. Respiratory muscle strength in patients with HF have been found to be significantly correlated to peak VO_2_ and is an independent predictor of survival^[4]^. In rats with HF after induction of MI, the diaphragm has reduced strength and function^[3]^, however, the cellular processes leading to muscle dysfunction remain poorly understood.

In normal rats, Bisschop et al.^[12]^ verified that RMT leads to hypertrophy in IIa and IIb fiber types in the diaphragm. In our study, we observed no difference in hemodynamic function, morphological characteristics and CS activity after RMT in sham group, which can be partially explained by the fact that sham rats do not present peripheral muscles alterations, such as in the diaphragm, due to preserved cardiac and hemodynamic function. In addition, the intensity of RMT used in this study was likely insufficient to produce improvements in sham rats. Further studies should therefore be conducted to test different RMT protocols and intensities in healthy rats.

Lower cardiac output, reduction of muscle blood flow, and consequently impairment O_2_ transport to skeletal muscle promotes disorders at the cellular level in HF^[1,2]^, including the diaphragm muscle. In this context, alterations in energy metabolism play a substantial role in the functional defects such as decreased cardiac contractility, and decreased contraction and resistance to fatigue of the skeletal muscle. These alterations are encountered in cardiac muscle as well as in skeletal muscles and the diaphragm, and thus led to the proposal of a metabolic myopathy in HF^[19]^.

In canine and rat HF models, marked reductions in the complexes of the respiratory chain have been described in both cardiac and skeletal muscles^[20,21]^, which may sustain the abnormalities of cardiopulmonary function and its interactions and blood flow redistribution. Muscle oxidative capacities have been assessed by measuring respiration of mitochondria in permeabilized fibers with no limitation of substrates, ADP or oxygen^[22]^. The main mitochondrial pathways were investigated by measuring activity of key enzymes: CS, an enzyme of the Krebs cycle, COX, the complex IV of the respiratory chain, and mi-CK, an intermembrane space enzyme involved in energy transfer between mitochondria and cytosol in oxidative muscles^[19]^. Garnier et al.^[19]^ describe a significant mitochondrial dysfunction and altered biochemical markers in HF rats, with CS significantly reduced in the LV and soleus showing a lower sensitivity of muscle to HF. Mitochondrial marker enzyme activities in fresh muscle homogenate were measured in skeletal muscle in dogs with HF^[6]^ and CS was significantly decreased, suggesting a lower mitochondrial content^[6]^.

To better explore the relationship between beneficial changes of diaphragm oxidative and hemodynamic function due to RMT in HF, further investigations of the respiratory muscle performance, as well as other key metabolic and mitochondrial enzymes and complexes, not performed in our study, are needed. Histological evaluation of the adaptations in the lungs or diaphragm can also elucidate morphological alterations in response to RMT in HF rats.

In the present study, we found that CS activity of the diaphragm in HF rats lower by 61% compared with the Sed-Sham group. After a 6-week RMT protocol, HF rats has a 142% increase in CS activity. Furthermore, we observed a significant correlation between CS activity and hemodynamic function, as demonstrated by a negative correlation between CS and LVEDP (r=-0.60, *P*=0.01) and positive correlation between CS and LVSP (r=0.46, *P*=0.05).

Additionally, to our knowledge, this study was the first to observe a beneficial change in oxidative metabolism after RMT in HF rats. These diaphragmatic muscle changes observed here with RMT were consistent with those elicited by endurance training of the limb muscles in normal subjects. These potential adaptations caused by RMT on the diaphragm in HF rats could be explained by some physiological consequences including: 1) an increase in the maximum speed of contraction^[23]^; (2) an attenuation of microvascular oxygen delivery-to-utilization mismatch; and, consequently, 3) a decrease in fatigability^[24]^.

### Clinical Implications

CABG is recommended in patients with HF and left ventricular systolic dysfunction and promotes benefits on cardiovascular mortality^[8]^. The 10-years follow-up of the STICH (Surgical Treatment for Ischemic Heart Failure) trial demonstrated a reduction in all-cause mortality in patients with HF who received CABG associated to medical therapy compared with medical therapy alone^[8]^. However, PPCs following CABG surgery, such as respiratory weakness, can influence on morbidity, mortality, and length of hospital stay^[15]^.

It has been postulated that RMT increases inspiratory muscle strength and endurance in patients with HF, which translates into marked improvement in exercise tolerance and quality of life^[14]^. Moreover, preoperative^[16]^ and postoperative^[15]^ RMT improves functional capacity submaximal, inspiratory muscle strength and decreases PPCs in patients undergoing cardiac surgery^[15,16]^.

Hulzebos et al.^[9]^ demonstrated that preoperative RMT in patients undergoing CABG surgery, which include patients with HF secondary to ischemic cardiomyopathy (left ventricular ejection fraction < 40%), reduced the incidence of PPCs (by 50% compared with patients receiving usual care), and, consequently, reduced the duration of postoperative hospitalization.

Diffuse parenchymal processes, and cardiogenic and noncardiogenic pulmonary edema are among the causes of acute respiratory failure after cardiac surgery^[25]^. Pulmonary edema is related with reduced pulmonary and functional capacity; however, the reduction of pulmonary edema decreases CPPs, including in patients undergoing CABG surgery. In this context, experimentally, our group has previously demonstrated that RMT decreased pulmonary edema and, consequently, improves respiratory mechanics in HF rats^[7]^. In this study, rats with HF presented less pulmonary congestion (*P*<0.0001) for training effect. We believe that these results could contribute to a better understanding of these potential mechanisms in the improvement of pulmonary and functional capacity with decreased pulmonary edema.

## CONCLUSION

RMT produces beneficial adaptations on the diaphragm muscle, which is linked to improvements in LV hemodynamics and blood pressure in HF. Additionally, diaphragm oxidative improvements translate into the prevention of RV hypertrophy and lung congestion, possibly blunting the severe progression of HF post-MI in rats.

**Table:** 

Authors' roles & responsibilities	
RBJ	Conception and design study; manuscript redaction or critical review of its content; final manuscript approval
MB	Conception and design study; manuscript redaction or critical review of its content; final manuscript approval
ABS	Conception and design study; manuscript redaction or critical review of its content; final manuscript approval
RA	Conception and design study; manuscript redaction or critical review of its content; final manuscript approval
PDL	Conception and design study; manuscript redaction or critical review of its content; final manuscript approval
